# Instant Typing Is Essential to Detect Transmission of Extended-Spectrum Beta-Lactamase-Producing *Klebsiella* Species

**DOI:** 10.1371/journal.pone.0136135

**Published:** 2015-08-28

**Authors:** Anne F. Voor in 't holt, Juliëtte A. Severin, Wil H. F. Goessens, René te Witt, Margreet C. Vos

**Affiliations:** Department of Medical Microbiology and Infectious Diseases, Erasmus MC University Medical Center, Rotterdam, The Netherlands; Institut Pasteur, FRANCE

## Abstract

**Background:**

Infections with multidrug-resistant (MDR) microorganisms are an increasing threat to hospitalized patients. Although rapid typing of MDR microorganisms is required to apply targeted prevention measures, technical barriers often prevent this. We aimed to assess whether extended-spectrum beta-lactamase (ESBL)-producing *Klebsiella* species are transmitted between patients and whether routine, rapid typing is needed.

**Methods:**

For 43 months, the clonality of all ESBL-producing *Klebsiella* isolates from patients admitted to Erasmus MC University Medical Center in Rotterdam, the Netherlands was assessed with Raman spectroscopy. A cluster was defined as n ≥2 patients who had identical isolates. Primary patients were the first patients in each cluster. Secondary patients were those identified with an isolate clonally related to the isolate of the primary patient.

**Results:**

Isolates from 132 patients were analyzed. We identified 17 clusters, with 17 primary and 56 secondary patients. Fifty-nine patients had a unique isolate. Patients (*n* = 15) in four out of the 17 clusters were epidemiologically related. Ten of these 15 patients developed an infection.

**Conclusions:**

Clonal outbreaks of ESBL-producing *Klebsiella* species were detected in our hospital. Theoretically, after Raman spectroscopy had detected a cluster of *n* ≥2, six infections in secondary patients could have been prevented. These findings demonstrate that spread of ESBL-producing *Klebsiella* species occurs, even in a non-outbreak setting, and underscore the need for routine rapid typing of these MDR bacteria.

## Introduction

Infections with multidrug-resistant (MDR) microorganisms are an increasing threat to hospitalized patients, leading to high morbidity and mortality because of ineffective antibiotic treatment [1, 2]. In general, carriage of antimicrobial resistant organisms occurs *de novo* by induction and selection during therapy (endogenous sources) or by transmission of already resistant organisms (exogenous sources). In healthcare settings transmission occurs either direct—patient to patient—or indirect via surrounding reservoirs or sources in the environment.[3] Exogenous infections can be prevented using measures aiming at preventing transmission. Nevertheless, Gram-negative bacteria producing beta-lactamase enzymes such as extended-spectrum beta-lactamase (ESBL) or carbapenemases are currently of major concern. These resistant bacteria are a major cause of healthcare-related infections, especially in patients with a prolonged hospital stay [4].

Although the spread of ESBL-producing *Klebsiella* spp. has not yet been elucidated, current data indicate that they are mainly polyclonal with some small clusters in the hospital [5, 6]. However, if these small clusters go unnoticed and/or appropriate infection control measures are not taken, they may result in large hospital-wide outbreaks [7]. Because the prevalence of ESBL-producing *Klebsiella* spp. is increasing worldwide, hospitals must remain vigilant [4, 8]. It should however be noted that the prevalence differs among patient groups, clinical and geographic settings [4, 8].

Rapid typing of MDR microorganisms can be of great support to demonstrate spread of related microorganisms. As a result targeted infection prevention measures are to be applied to stop transmission. Although MDR bacteria can be easily detected in a routine setting using proper indicator antibiotics combined with confirmation assays, rapid typing of these isolates is often not routinely performed. This is mainly due to technical barriers: most typing techniques are time consuming and laborious and therefore difficult to implement in routine diagnostics. Raman spectroscopy is a rapid technology that is used for whole organism fingerprinting and is applied in microbiological laboratories. This technique provides highly information-rich spectra which are required for maximum discriminatory power to distinguish unrelated microorganisms—which is required in outbreak management [9–11].

In this study, we applied this rapid typing technique to all ESBL-producing *Klebsiella* spp. that were identified in our hospital in the Netherlands for 43 months, in order to answer the following questions: first, are ESBL-producing *Klebsiella* spp. transmitted between patients? Second, is routine, rapid typing needed in an apparently non-outbreak setting?

## Materials and Methods

### Ethics statement

Screening was performed as part of the infection control strategy using non-invasive sampling. The microbiological and epidemiological analyses were in first instance performed to develop new strategies for infection control. Also, according to the Dutch regulation for research with human subjects, neither medical nor ethical approval was required to conduct the study since the data were retrospectively recorded. However, we received approval from the medical ethics research committee of the Erasmus University Medical Center (Erasmus MC) in Rotterdam, the Netherlands to conduct this study (MEC-2011-085). The existing data from the electronic medical records could not be recorded anonymously as the patient name is always present. Before data analyses however, the opt-out list available at our department was consulted and patient names were removed from the dataset by A.F. Voor in ‘t holt. The authors had no direct interaction with the patients and no new patient data were collected during the study period.

### Population

This study was conducted at the Erasmus MC in Rotterdam, the Netherlands. This is a 1,320-bed university hospital, with 97 beds in the adult or pediatric intensive care unit (ICU) (Erasmus MC, 2012). All medical specialties are available. Table 1 presents the number of hospital admissions from 1 January 2010 until 1 August 2013, the number of patients identified with *Klebsiella* spp.—susceptible and ESBL-producing isolates—and the number of patients who were included in the current study.

**Table 1:** Study characteristics.

	2010	2011	2012	2013^a^
No. of hospital admissions	40,626	41,773	41,001	21.893
No. of clinical admission days	292.209	288.799	299.736	n.a.
*Klebsiella pneumoniae* ^b^	774	677	619	320
ESBL-producing *K*. *pneumoniae* ^c^	54 (40)	47 (10)	62 (48)	34 (25)
ESBL-rate per 1000 hospital admissions	1,33	1,13	1,51	1,55
*Klebsiella oxytoca* ^b^	370	338	351	161
ESBL-producing *K*. *oxytoca* ^c^	7 (0)	5 (3)	15 (5)	3 (1)
ESBL-rate per 1000 hospital admissions	0,17	0,12	0,37	0,14

Abbreviations: n.a., not available, no., number.

^a^1 January until 1 August 2013;

^b^One per patient;

^c^Between brackets: number of patients included in current study

### Study Design and Data Collection

We included patients of all departments with a microbiologically confirmed ESBL-producing *K*. *pneumoniae* or *K*. *oxytoca* between 1 January 2010 and 1 August 2013. After detection, all patients were immediately managed in contact isolation and placed in single-occupancy rooms. Contact isolation included the use of gloves and gowns, and disinfection of hands and wrists with hand alcohol when entering and leaving the room. No active surveillance and/or contact investigation were carried out. Isolates were cultured from clinical samples, either because of 1) assumed infection or 2) surveillance purposes in the ICU and hematology departments—patients receiving selective digestive tract decontamination are routinely tested for the presence of (resistant) Gram-negative bacteria twice weekly. This study consisted of two study periods. During study period I, 1 January 2010 until 1 September 2012, isolates were collected and typed retrospectively. Also, clinical data were collected retrospectively. However, carbapenemase-producing isolates were immediately typed after detection at that time. During study period II (1 September 2012 until 1 August 2013), typing with Raman spectroscopy was performed immediately after detection and clinical data were collected prospectively. In this report, data from both study periods were combined. Preventive measures were equal in both periods.

For each first isolate, we recorded patient data and bacteriological data, which were obtained from electronic patient records. Age of the patient was defined as age at day of detection of the first ESBL-producing *Klebsiella* isolate. Mortality was defined as death from any cause within one year after the day of detection of the first ESBL-producing *Klebsiella* isolate [12].

To identify healthcare-related infections and the specific type of infection, we used the criteria published by the Centers for Disease Control and Prevention (CDC) [13]. We investigated microbiological data and medical records of included patients to distinguish colonization from infection.

### Microbiological Analysis

Cultures were performed at the diagnostic laboratory of the department of Medical Microbiology and Infectious Diseases (Erasmus MC, Rotterdam, The Netherlands). In study period I, bacteria were taken from frozen stock cultures kept at -80°C, and were subsequently analyzed with Raman spectroscopy. In study period II, bacteria were typed with Raman spectroscopy immediately after the initial culture.

Identification and susceptibility testing for *K*. *pneumoniae* and *K*. *oxytoca* were performed using VITEK 2 (bioMérieux, Lyon, France), and results were interpreted according to the EUCAST clinical breakpoints. ESBL confirmation was performed with either the combination disk-diffusion test (Rosco Diagnostica, Taastrup, Denmark) or the Etest (bioMérieux, Lyon, France). *K*. *oxytoca* isolates were regarded as ESBL-producing when resistant to ceftazidime and demonstrating synergy between ceftazidime and clavulanic acid. *K*. *oxytoca* isolates were regarded as hyperproducers of K1 (KOXY) chromosomal beta-lactamase if they showed resistance to cefuroxime, piperacillin-tazobactam and aztreonam, borderline resistance to cefotaxime and cefepime, but remained susceptible to ceftazidime [14]. Presence of carbapenemases was confirmed using real-time PCR for *bla*_IMP_, *bla*_KPC_, *bla*_NDM_, *bla*_OXA-48_, and *bla*_VIM_ genes [15].

### Clonal Relatedness

Clonal relatedness was investigated with Raman spectroscopy using SpectraCell*RA* analysis (SC*RA*). Cultures, sample preparation, and SC*RA* measurements were performed according to the operators manual (version 1.7) [16]. Analyses and calculations were performed as described previously [17]. A cluster was defined as *n* ≥2 patients who had identical isolates as indicated with Raman spectroscopic analysis. A distinction was made between primary and secondary patients, and patients with a unique isolate. A primary patient was defined as the first patient in time in a cluster. Secondary patients were all subsequent patients who had a proven clonal relationship with the primary patient. Unique was defined as patients with a non-cluster isolate. When analyzing, data from primary patients and from patients with a unique isolate were combined since they were both the first patient in time with a certain type. The only difference was that primary patients generated secondary patients, and patients with a unique isolate did not.

### Spatial Analysis

To investigate if patients identified in Raman spectroscopic clusters were epidemiologically related, four different definitions of epidemiological relatedness were created from highest to lowest likelihood (Table 2).

**Table 2:** Definitions of epidemiological relatedness.

	Definitions			
	Definite	Probable	Possible	Impossible
Same patient room	yes	yes	No	no
Same department	yes	yes	Yes	no
Same period	yes	maybe^a^	maybe^b^	maybe^c^

^a^Same patient room within 3 months after primary patient has left;

^b^Same department within 3 months after primary patient has left;

^c^Same period but not the same department or patient room

### Statistical Analysis

We calculated the transmission index (TI) to analyze the transmission dynamics using two different formulas. Firstly, it was calculated as the number of secondary patients divided by the number of primary patients and the number of patients with a unique isolate—with the results of Raman spectroscopy. Secondly, as the number of secondary patients who were epidemiologically related to the primary patient (definitions ‘definite’ and ‘probable’ combined, Table 2) divided by the number of primary patients and the number of patients with a unique isolate.

Basic patient characteristics (e.g. age, gender, death from any cause within one year after positive culture) were analyzed as percentages and means using Microsoft Excel 2010.

## Results

### Identification and characteristics of included patients

**Fig 1:**
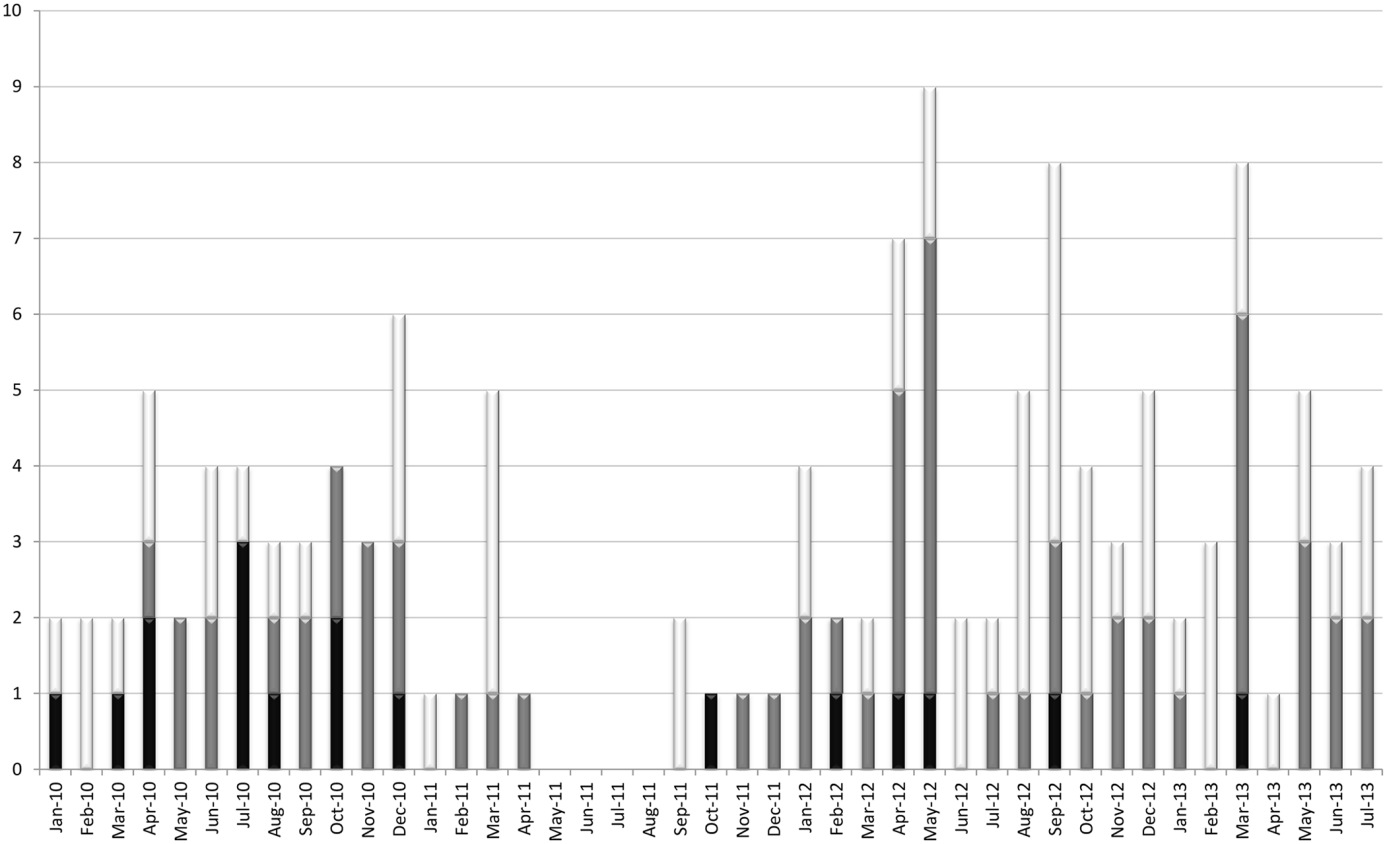
Distribution of patients identified with an ESBL-producing *Klebsiella* spp. (*n* = 132), January 2010 until August 2013. Black = primary patients (*n* = 17); the first patient in time in a cluster, identified with Raman spectroscopy. Grey = secondary patients (*n* = 56); all subsequent patients who had a proven clonal relationship with the primary patient. Light grey = patients with a unique isolate (*n* = 59).

For the period from 1 January 2010 until 1 August 2013 we included 132 patients with an ESBL-producing *Klebsiella* isolate (Table 3, Fig 1). ESBL-producing *K*. *pneumoniae* was cultured in 123 patients and ESBL-producing *K*. *oxytoca* was cultured in nine patients (Table 3). Ninety isolates were obtained from clinical samples, and 42 isolates were obtained from surveillance cultures. In total, 17 clusters were identified with Raman spectroscopy, comprising 73 patients (cluster size ranging from two to ten patients), and 59 patients were identified with a unique isolate. Among the 73 patients with a cluster isolate, we identified 17 primary patients, and 56 secondary patients (Table 3). Eighty-six out of 132 patients (65.2%) developed an infection with an ESBL-producing *Klebsiella* spp. (Table 3). Fifty-one out of 17 primary plus 59 unique patients (67.1%) and 35 out of 56 secondary patients (62.5%) developed or had an infection with the ESBL-producing *Klebsiella* spp.

**Table 3:** Characteristics of clusters and unique isolates as defined with Raman spectroscopy, January 2010 until August 2013.

	Total no. of patients	No. of primary patients^a^		No. of secondary patients	
		Infection	Colonization	Infection	Colonization
Total	132	51	25	35	21
*K*. *pneumoniae*	123	50	22	33	18
*K*. *oxytoca*	9	1	3	2	3
Cluster 1	2	0	1	1	0
Cluster 2	4	1	0	0	3
Cluster 3	9	1	0	5	3
Cluster 4	10	0	1	8	1
Cluster 5	5	0	1	4	0
Cluster 6	5	0	1	2	2
Cluster 7	3	1	0	1	1
Cluster 8	3	0	1	1	1
Cluster 9	2	0	1	1	0
Cluster 10	10	1	0	5	4
Cluster 11	2	0	1	1	0
Cluster 12	2	0	1	1	0
Cluster 13	5	1	0	2	2
Cluster 14	3	1	0	2	0
Cluster 15	2	1	0	0	1
Cluster 16	3	1	0	1	1
Cluster 17	3	0	1	0	2
Unique isolates	59	43	16	n.a.	n.a.

Abbreviations: n.a., not applicable, no., number.

^a^Including patients with a unique isolate

Eight patients were identified with an ESBL-producing *K*. *pneumoniae* isolate that was also resistant to imipenem and/or meropenem and three patients with an ESBL-producing *K*. *pneumoniae* isolate intermediately susceptible to imipenem and meropenem. The *bla*_KPC_ gene was detected in six out of these 11 patients, the *bla*_OXA-48_ gene was demonstrated in isolates of three patients and the *bla*_NDM_ gene was present in isolates of two patients. According to Raman spectroscopic analyses, seven out of these 11 patients had a unique isolate. In cluster 15 (*n* = 2), both patients had a KPC-producing isolate. In cluster ten (*n* = 10), two patients had an NDM-producing isolate.

### Clinical Epidemiology

From all 132 patients with an ESBL-producing *Klebsiella spp*. isolate, 130 were residents of the Netherlands. The median age of these 132 patients was 57.4; 58.3 for primary patients plus patients with a unique isolate (*n* = 17 + *n* = 59) and 54.8 for secondary patients (*n* = 56). Two clusters (cluster 6 and 11) consisted of newborns only. The overall mortality rate one year after detection of the bacteria was 17.4% (*n* = 23), consisting of three primary and nine secondary patients, and 11 patients with a unique isolate. Overall male percentage was 62.1%; 61.8% in primary patients plus patients with a unique isolate and 62.5% in secondary patients. Twenty-six patients (19.7%) were organ transplant recipients; three primary and eight secondary patients, and 15 patients with a unique isolate. The majority of patients received a kidney allograft (*n =* 22). Basic patient characteristics are displayed in Table 4.

**Table 4:** Clinical characteristics of patients infected or colonized with ESBL-producing *Klebsiella* spp. and clinical characteristics of primary and secondary patients.

Variables	No. of patients (%)		No. of patients (%)	
	Infection (*n* = 86)	Colonization (*n* = 46)	Primary^b^ (*n* = 76)	Secondary (*n* = 56)
Gender, male	61 (70.9)	21 (45.7)	47 (61.8)	35 (62.5)
Age, mean years (SD)	53.4 (24.0)	42.9 (29.7)	52.3 (23.4)	46.3 (30.1)
COPD	6 (7.0)	1 (2.2)	5 (6.6)	2 (3.6)
1-year mortality^a^	18 (20.9)	5 (10.9)	14 (18.4)	9 (16.1)
Organ transplantation	17 (19.8)	9 (19.6)	18 (23.7)	8 (14.3)
No. of primary patients^b^	51 (59.3)	25 (54.3)	n.a.	n.a.
No. of patients who had an infection	n.a.	n.a.	51 (67.1)	35 (62.5)

Abbreviations: COPD, chronic obstructive pulmonary disease, SD, standard deviation, no., number, n.a., not applicable.

^a^Death from any cause within one year after the first positive culture;

^b^Including patients with a unique isolate

The most frequent specimen containing ESBL-producing *Klebsiella* spp. cultures for all patients was urine (45.5%), followed by rectum samples (21.2%). In primary patients plus patients with a unique isolate, 73.0% of patients who had a positive urine sample developed or had an infection, compared with 47.8% of secondary patients (*P* value 0.049). Twenty-three percent of primary patients who had a positive rectum sample developed or had an infection, compared with 53.3% of secondary patients (*P* value 0.102).

### Spatial Analysis

From the 132 patients, 95 were admitted to the hospital to 36 different departments. Thirty-seven patients were not admitted when the ESBL-producing *Klebsiella* isolate was detected. Also, 31 patients (23.5%) were admitted to either the ICU or the hematology department.

**Fig 2:**
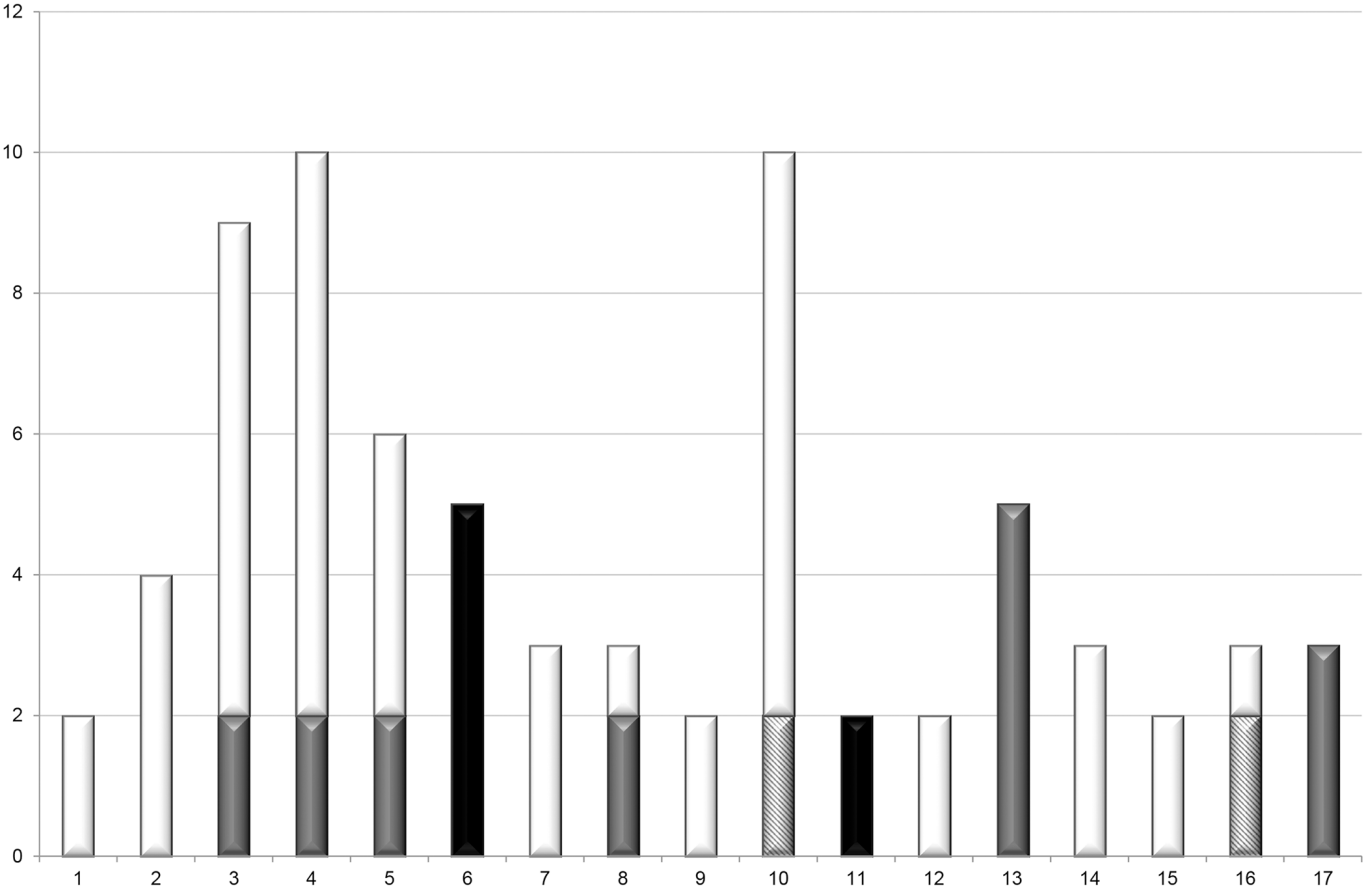
Epidemiological relatedness of patients in the 17 clusters. Y-axis: number of patients, x-axis: cluster number. Black = definite, grey = probable, wide downward diagonal lines = possible, white = impossible.

Patients in cluster 6 (*n* = 5) and 11 (*n* = 2) were, according to our definitions, definitely related as patients were related in time and place (Fig 2). Cluster 6 and 11 both consisted of newborns infected (*n =* 3*)* or colonized (*n =* 4*)* with *K*. *oxytoca* bacteria (Table 5). Patients in cluster 13 (*n* = 5) and 17 (*n* = 3) were probably related. In six clusters (cluster 3, 4, 5, 8, 10 and 16) a more diverse picture of epidemiological relatedness was found (Table 5, Fig 2). Patients in cluster one (*n* = 2), two (*n* = 4), seven (*n* = 3), nine (*n* = 2), 12 (*n* = 4), 14 (*n* = 3) and 15 (*n* = 2) could not be related to each other in time and place: transmission in the hospital was impossible according to the information in the medical records of the patients and our definitions (Fig 2).

**Table 5:** The number of infected and colonized patients in the eight clusters identified with Raman spectroscopy with a definite or probable epidemiological relationship.

Cluster no.	Definite			Probable		
	Infection		Colonization	Infection		Colonization
	Prim	Sec		Prim	Sec	
Cluster 3	0	0	0	1	1	0
Cluster 4	0	0	0	0	1	1
Cluster 5	0	0	0	0	2	0
Cluster 6	0	2	3	0	0	0
Cluster 8	0	0	0	0	1	1
Cluster 11	0	1	1	0	0	0
Cluster 13	0	0	0	1	2	2
Cluster 17	0	0	0	0	0	3

Abbreviations: no., number; prim, primary patients including patients with a unique isolate; sec, secondary patients. Epidemiological relatedness is presented as number of patients.

When using the results of Raman spectroscopy, the transmission index was 0.74 (56 divided by 17 plus 59; definition 1). When dividing epidemiologically related secondary patients in clusters by all primary patients, the transmission index declined to 0.30 (23 divided by 17 plus 59; definition 2).

## Discussion

### Summary of Evidence

There are many ways of preventing antibiotic resistance, and especially the spread of antibiotic-resistant bacteria can be prevented in many different ways. A powerful policy is to prevent transmission of these resistant bacteria from patient to patient, the success of which depends on the speed and accuracy of the typing method used for early identification of related clusters. Rapid and accurate typing allows targeted infection control measures to be applied immediately, thereby preventing morbidity and mortality among patients and reducing hospital costs [18].

In the present study, we performed an in-depth analysis of the epidemiology of ESBL-producing *Klebsiella* spp. in a large university hospital using clinical samples and screening cultures over a 43-month period. Our results demonstrate that clonal outbreaks with ESBL-producing *Klebsiella* spp., as confirmed epidemiologically and by Raman spectroscopy, definitely occurred in our hospital and should have been prevented. In theory, as soon as clonality had been demonstrated using Raman spectroscopy (*n* ≥2), six out of ten infections in seven different clusters could have been prevented by implementing immediate additional infection control measures. These additional infection control measures include immediate disinfection of the patient rooms and sanitation involved and a contact investigation among roommates. The contact investigation is performed from the first admission date of the primary patient until the date of contact isolation or discharge of the last secondary patient. The possible (NDM) and impossible (KPC) transmissions of carbapenemase-producing *Klebsiella* spp. in cluster 10 and cluster 15 were noted with typing at the time of detection. Additional screening of contact patients was performed, which did not reveal other colonized patients. Previous reports on ESBL-producing *Klebsiella* spp. have shown similar results, although some studies have identified large clusters [19]. However, these studies often did not investigate whether patients in clusters—identified by molecular analysis—were also epidemiologically related [19].

Due to the retrospective nature of our analysis, our results represent merely the tip of the iceberg. We hypothesize that if patients had been routinely screened for MDR bacteria upon admission, during admission, upon discharge and/or after the finding of a primary case, we would have found more and larger clusters in which patients were epidemiologically related. The fact that we were not able to define relatedness in all secondary cases was presumably due in part to unidentified carriers (missed cases) and transmission. A possible additional theoretical explanation of secondary cases is community spread of these type of bacteria, as carriage in the community has been reported in the literature and is of rising concern. However, in the Netherlands spread in the community has not been reported so far [20].

The transmission index was 0.74. When we used epidemiological relatedness as a criterion for secondary patients, the transmission index decreased to 0.30. However, the R_0_ could not be calculated as each patient identified with an ESBL-producing *Klebsiella* spp. isolate was nursed in contact isolation and placed in a single-occupancy room immediately after detection. At that moment it was not clear whether this patient was a primary or a secondary patient. These prompt measures may have kept the transmission index low.

### Limitations

Our study has a number of limitations. First, patients were not routinely cultured for carrying multi-resistant Enterobacteriaceae at admission or discharge during the study periods in our hospital and we were not able to include all the carriers identified in our hospital (Table 1). The presence of unidentified carriers therefore cannot be ruled out, and we may have underestimated the number of affected patients. Second, the surveillance cultures in our dataset were only obtained from ICU and hematology patients while the remaining cultures were obtained from clinical samples. Third, in seven of the 17 clusters identified using Raman spectroscopy no epidemiological relatedness between patients was found. This may be due to low numbers of cultures—thus missing cases—and/or to our definition of epidemiological relatedness that may have been too strict (Table 2). Since contamination of the environment as a source needs to be taken into consideration when studying transmission, we chose a 3-month window during which we still considered transmission to be possible (Table 2) [21, 22]. Unfortunately, neither nationwide guidelines nor even the CDC provide a definition of epidemiological relatedness. Finally, we did not include the identification of the specific ESBL genes (e.g. *bla*_CTX-M_, *bla*_SHV_, *bla*_TEM_) and plasmid types in our analysis of the 132 isolates. Therefore, transmission between different strains of plasmids carrying ESBL genes cannot be ruled out.

### Conclusion and Clinical Implication

In summary, as transmission occurred during the entire study period and throughout the entire hospital, there is an urgent need for instant typing to detect and subsequently prevent the spread of ESBL-producing *Klebsiella* spp. We conclude that typing should continuously be performed, also in an apparent non-outbreak situation. In order to relate those patients who have clonally clustered isolates, it is important to actively screen contact patients for carriage of ESBL-producing *Klebsiella* spp. These recommendations are similar to those given in previous investigations and in the recent Dutch MDRO guideline [19, 23–26]. A rapid typing method that is easy to implement and that can be used for the early identification of clusters is Raman spectroscopy. Through the implementation of immediate infection-prevention strategies, those small outbreaks identified early on can be stopped and further transmission can be prevented. However, further research is needed to investigate the presence of ESBL-producing *Klebsiella* spp. and their transmission dynamics in other hospital settings. This will give further insight in the transmission magnitude of *Klebsiella* spp. in the hospital.
